# Understanding the Social Context of the ASGM Sector in Ghana: A Qualitative Description of the Demographic, Health, and Nutritional Characteristics of a Small-Scale Gold Mining Community in Ghana

**DOI:** 10.3390/ijerph121012679

**Published:** 2015-10-12

**Authors:** Rachel N. Long, Elisha P. Renne, Niladri Basu

**Affiliations:** 1 Department of Environmental Health Sciences, University of Michigan School of Public Health, 1415 Washington Heights, Ann Arbor, MI 48109, USA; E-Mail: rachlong@umich.edu; 2 Department of Afroamerican and African Studies, Department of Anthropology, University of Michigan, 101 West Hall, Ann Arbor, MI 48109, USA; E-Mail: erenne@umich.edu; 3 Faculty of Agricultural and Environmental Sciences, McGill University, CINE Building, Macdonald Campus of McGill University, 21,111 Lakeshore Rd., Ste. Anne de Bellevue, QC H9X 3V9, Canada

**Keywords:** gold mining, artisanal and small-scale mining, ASGM, Ghana, public health, demographic health, survey, census

## Abstract

This descriptive paper describes factors related to demographics and health in an artisanal and small-scale gold mining (ASGM) community in Ghana’s Upper East Region. Participants (n = 114) were surveyed in 2010 and 2011, adapting questions from the established national Demographic Health Survey (DHS) on factors such as population characteristics, infrastructure, amenities, education, employment, maternal and child health, and diet. In the study community, some indicators of household wealth (e.g., radios, mobile phones, refrigerators) are more common than elsewhere in Ghana, yet basic infrastructure (e.g., cement flooring, sanitation systems) and access to safe water supplies are lacking. Risk factors for poor respiratory health, such as cooking with biomass fuel smoke and smoking tobacco, are common. Certain metrics of maternal and child health are comparable to other areas of Ghana (e.g., frequency of antenatal care), whereas others (e.g., antenatal care from a skilled provider) show deficiencies. Residents surveyed do not appear to lack key micronutrients, but report lower fruit and vegetable consumption than other rural areas. The results enable a better understanding of community demographics, health, and nutrition, and underscore the need for better demographic and health surveillance and data collection across ASGM communities to inform effective policies and programs for improving miner and community health.

## 1. Introduction

Artisanal and small-scale mining (ASGM) has expanded in many regions of the world, and is estimated to employ over 20 million people worldwide, with about 100 million individuals economically dependent upon the sector [1]. Gold from small-scale mines may account for 20% to 30% of the world's output [2]. Ghana, formerly known as “the Gold Coast,” is one of the most important gold producing countries in the world, and is second only to South Africa in production from the African continent [3]. Though gold has been mined in Ghana for over 1000 years, there has been tremendous recent growth in the sector, with a sevenfold increase in gold production between 1980 and 2000 [4]. As of 2013, gold accounts for 34.4% of Ghana’s national exports [5]. ASGM accounts for 10.5% of Ghana’s national gold production, and employs between 500,000 and 1 million people in Ghana, predominantly in rural areas [6,7]. 

Worldwide, poverty drives ASGM activity. In Ghana, 38% of the population lives below the national poverty line, and in rural areas the rate of poverty amongst agricultural communities can be as high as 65% [8]. As a low-tech, labor-intensive industry with few barriers to entry, ASGM has become an alluring alternative livelihood for rural Ghanaians [9]. Widely practiced in the country’s southwest region, it has recently expanded to the north where gold is more difficult to extract but nonetheless viable. In Ghana’s Upper East Region, ASGM has grown rapidly over the past 20 years due to unemployment in cities, deregulation of gold mining in Ghana, proliferation of industrial mining activities, and the increasing price of gold worldwide [10]. As of 2010, it is estimated that over 10,000 men, women, and children are employed directly in the district’s small-scale gold mines, making them an economic cornerstone of the region [11].

A variety of public health concerns have accompanied the expansion of ASGM in Ghana’s Upper East Region. These concerns include, for example, water contamination, lack of sanitation facilities and infrastructure, inhalation of dust from pulverized ore, lack of protective equipment, and exposure to mercury and other heavy metals [12,13]. Public health research on ASGM communities has focused predominantly on exposure to mercury and other heavy metals [14,15,16,17,18], yet little has been published about other factors unique to these sites that may contribute to (or detract from) community health and well-being. For example, physical aspects of mining communities such as local infrastructure and sanitation facilities, amenities, and water supply remain uncharacterized. Social, behavioral, and economic factors such as population demographics, education level, employment, diet and nutritional status, health care access, tobacco usage, and variables related to maternal and child health are also largely unknown. Such census data is crucial to informing and evaluating public health actions. The goal of this paper is to provide a descriptive, yet holistic, overview of key demographic, health, and nutritional factors in a legally registered ASGM concession known as Kejetia in Upper East, Ghana. This qualitative description offers background information to support the reader's understanding of the context for the journal articles included in this special issue on ASGM in Ghana (http://www.mdpi.com/journal/ijerph/special_issues/asgm).

## 2. Methods

Kejetia, the ASGM community that is the focus of this study, is located in the Talensi district of Ghana’s Upper East region, and has an estimated population of 2500 as of 2010 [19]. Concession owners and traditional chiefs gave permission to work in the community. Approval was obtained from the University of Michigan Institutional Review Board (HUM00028444) concerning adult human subjects research. At the beginning of every interview, the study aims, objectives, risks, and benefits were explained to all participants in their preferred language (English, Fra Fra, Twi, Dagbani, Maprusi, or Hausa) with the assistance of a translator. 

In 2010, women to be interviewed were selected via convenience sampling. In 2011, all households in Kejetia were numbered, mapped, and grouped into 20 clusters of an average of 19.2 houses based on geographic proximity, using a handheld global positioning system (GPS; Oregon 450; Garmin International, Inc., Olathe, KS, USA). Each household was then assigned a number within its cluster, and households were selected by randomly pulling numbers from a bag. Up to three households were interviewed per day, each from a different cluster. Each cluster had two to three participating households in total. If a household was not eligible or declined participation, another number from within the cluster was pulled from the bag until an appropriate household was found. For the purposes of the survey, we adhered to the local definition of household, which is group of people who “eat from the same pot”. 

We conducted two surveys in Kejetia (May–June 2010 and May–July 2011). Questions in both surveys were adapted from the 2008 Ghana Demographic and Health Survey (Ghana DHS) [20]. Surveys were written in English and administered by a team of US researchers and local translators in each participant’s language of choice. The 2010 survey was conducted among 60 women living in Kejetia aged 15–48 with an average age of 26.8. The survey covered topics including reproductive histories and maternal and child health. In 2011, at each identified household, researchers requested to interview the head of the household. The household head or an identified alternative completed a survey on household demographics, characteristics, and amenities. A maximum of four adults per household, including the head of household or identified alternative, were administered a separate adult member survey, which included occupational history and smoking history. These adults, or a subset of these adults, in each household also completed a 24-hour recall survey on diet, and answered diet questions on behalf of dependent children in the household. In Kejetia, 26 of the interviewees were women and 28 were men. Interviewees’ ages ranged from 18 to 65 with an average age of 32. 

## 3. Results and Discussion

### 3.1. Household Population and Housing Characteristics

#### 3.1.1. Population Characteristics

**Figure 1:**
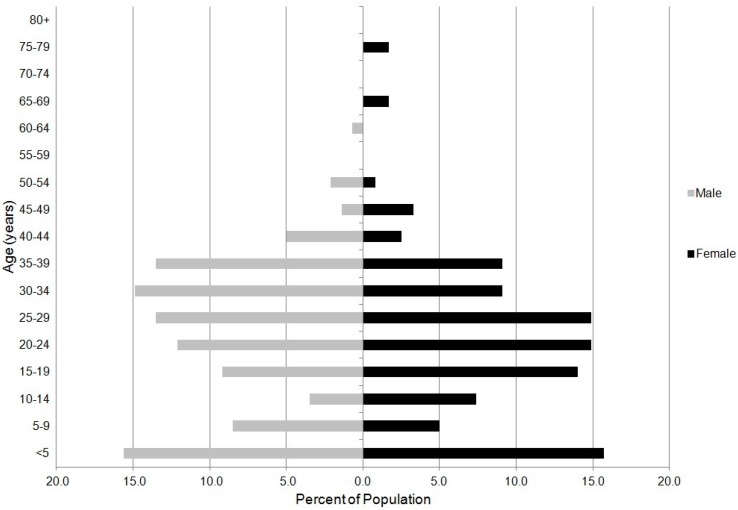
Demographic age group distribution in Kejetia based on 504 people sampled in 2011.

In Kejetia, 27.8% of the surveyed population is under 15 years of age, and about 1.6% of is above 65 years (Figure 1). Over two-thirds (68.8%) of the surveyed population is between 15 and 49, compared to 45.5% nationwide (via the 2008 Ghana DHS), which illustrates that Kejetia comprises mostly working-age individuals. Household size (number of people), which is an important factor in determining household well-being, is much higher on average in rural Ghana (4.0) than in urban Ghana (3.4) according to the 2008 Ghana DHS. Mean household size is larger in Kejetia (5.4) than in the rest of Ghana. The larger household size is likely reflective of the fact that many households contain either multiple unrelated workers or workers and their families (often including multi-generational extended families) rather than nuclear families. Further, 26.1% of households surveyed in Kejetia had three or more rooms for sleeping, compared to only 17.4% in rural Ghana and 12.0% in urban Ghana (Table 1).

**Table 1:** Percent distribution of household characteristics in Kejetia according to our 2011 demographic survey, and in urban Ghana, rural Ghana, and Ghana as a whole according to the 2008 Ghana DHS (Ghana Statistical Service 2009).

	Ghana DHS 2008			Survey 2011
	Urban	Rural	Total	Kejetia
**Flooring Material**				
Earth/sand	3.8	21.5	13.0	74.1
Dung	0.1	2.0	1.1	0.0
Wood/planks	0.1	0.0	0.1	0.0
Palm/bamboo	0.0	0.0	0.0	0.0
Parquet or polished wood	0.2	0.0	0.1	0.0
Ceramic tiles/terrazzo	5.0	0.7	2.7	0.0
Cement	56.0	65.3	60.8	25.9
Woolen carpet/synthetic carpet	18.3	3.6	10.6	0.0
Linoleum/rubber carpet	16.2	6.8	11.3	0.0
Other	0.3	0.0	0.1	0.0
**Total**	100.0	99.9	99.8	100.0
**Rooms used for sleeping**				
One	63.4	56.3	59.7	38.9
Two	23.8	26.0	24.9	35.2
Three or more	12.0	17.4	14.8	26.1
Missing	0.8	0.4	0.6	0.0
**Total**	100.0	100.1	100.0	100.2
Place for cooking **				
In the house	46.6	33.5	39.8	16.7
In a separate building	11.2	26.5	19.2	7.4
Outdoors	37.9	37.5	37.7	64.8
Other	NA	NA	NA	1.9
Missing	4.3	2.5	3.3	11.1
**Total**	100.0	100.0	100.0	101.9
**Cooking fuel**				
Electricity	0.9	0.2	0.5	0
LPG/natural gas/biogas	24	3.1	13.1	0
Kerosene	0.8	0.2	0.5	0
Charcoal	55.9	18.9	36.6	66.7
Wood	14.1	74.9	45.8	20.4
Straw/shrubs/grass	0.1	0.2	0.2	0
No food cooked in household	4.3	2.4	3.3	11.1
Missing	--	--	--	1.9
**Total**	100.1	99.9	100.0	100.1
Percentage using solid fuel *	70.1	94.0	82.6	94.0
Type of fire/stove among households using solid fuels *				
Closed stove/coal pot with chimney	0.1	0.2	0.2	0.0
Open fire/coal pot/open stove without chimney or hood	99.8	99.7	99.8	100.0
Missing	0.1	0.1	0.1	0.0
**Total**	100.0	100.0	100.1	100.0

** In Kejetia, respondents could give more than one answer, thus percentages can add up to more than 100; * Includes coal/lignite, charcoal, wood/straw/shrubs/grass, agricultural crops, and animal dung.

#### 3.1.2. Household Characteristics and Amenities

Household characteristics and amenities can help approximate a community’s general socioeconomic status. This is especially important in settings in which monetary income is not recorded as household goods can be used as a proxy for wealth [21]. Lower household socioeconomic status is associated with increased health vulnerability [22]. For example, the type of fuel used for cooking and the location of cooking can have an effect on respiratory health [23], and crowding and availability of sanitation facilities can influence the spread of communicable diseases [24]. Both our survey and the 2008 Ghana DHS asked respondents about housing materials and size, cooking fuels and methods, access to electricity, sources of drinking water, sanitation facilities, and ownership of durable goods (Table 1). To our knowledge, such household characteristics have not been well documented in ASGM communities in Ghana or elsewhere.

Most households surveyed in Kejetia have earth or sand floors (74.1%), whereas 60.8% of households surveyed in the 2008 Ghana DHS have cement floors (Table 1). Nearly two-thirds (64.8%) of households in Kejetia cook outdoors, compared to about 38% of households surveyed in the 2008 Ghana DHS. In Kejetia, 16.7% of households do at least some cooking in their house, and 7.4% do so in a separate building (Table 1). Charcoal is the cooking fuel used most in households in Kejetia (66.7%). Wood appears to be more commonly used in rural households (74.9%) surveyed in the 2008 Ghana DHS than in urban areas (14.1%), where charcoal is more common (55.9%). No households in Kejetia use LPG (liquid petroleum gas), natural gas, or biogas for cooking, whereas 13.1% of households surveyed elsewhere in Ghana do. Among houses using solid fuels (which includes coal, charcoal, wood, straw, shrubs, grass, agricultural residue, and animal dung), 100% in Kejetia use an open fire, open coal pot, or open stove. Nationwide, 99.8% of such households surveyed use an open fire, open coal pot, or open stove, and 0.2% use a closed stove or closed coal pot (Table 1).

**Figure 2:**
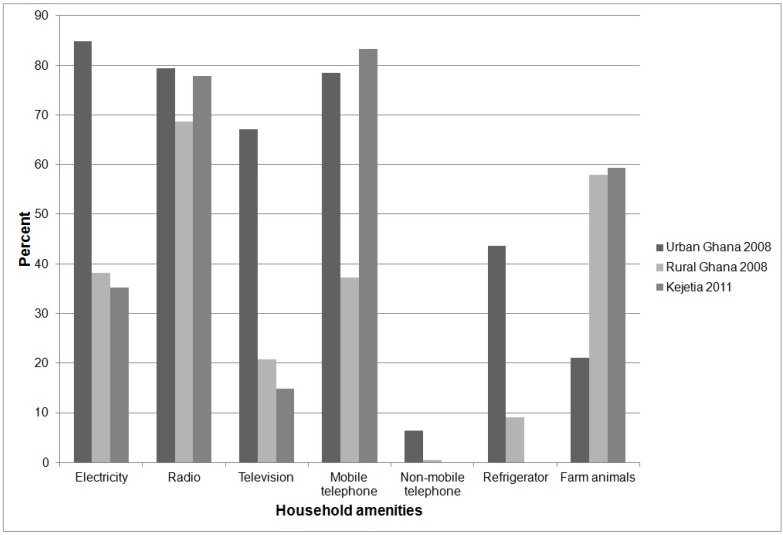
Chart of percent distribution of household amenities in Kejetia according to our 2011 demographic survey, and in urban Ghana and rural Ghana, according to the 2008 Ghana DHS.

Radios and televisions are not only an indicator of household wealth, but also an important means of communicating public health and other information. Functioning radios are as prevalent in Kejetia (77.8%) as in urban Ghana (77.8%) and more prevalent than in rural Ghana (68.6%) (Figure 2). Functioning televisions, however, are less common in Kejetia (14.8%) than in urban Ghana (67.1%), but their prevalence is comparable to that in other Ghanaian rural households (20.7%). 

The rate of mobile phone ownership among households surveyed is higher in Kejetia (83.3%) than in rural areas (37.3%), and comparable to that in urban areas (78.4%) (Figure 2). This may reflect nationwide changes in the rate of mobile telephone ownership in rural areas between 2008, when the last DHS occurred, and our survey, which occurred in 2011. No households surveyed in Kejetia reported owning a functioning refrigerator. Electricity is less common in Kejetia (35.2% of households) than in urban areas (84.8%) and rural areas (38.2%) of Ghana. We observed that all electricity in Kejetia seems to come from generators largely used for mining activities. Ownership of farm animals was slightly more common among households surveyed in Kejetia (59.3%) than in other rural areas (57.9%) and much more common than in urban areas (21.0%).

#### 3.1.3. Water

In urban areas across Ghana public taps/standpipes are the most common source of drinking water (39.2%). In rural Ghana, almost half of respondents reported using a tube well or borehole (47.8%) as their main source of drinking water. In Kejetia, however, the main drinking water source for most households is surface water (69.8%) (compared to only 9.7% of households nationwide) and the remainder of the respondents indicated using bottled or sachet water (30.2%). Kejetia shows a higher percentage of households using appropriate water treatment methods (11.1%) than elsewhere in Ghana (7.5%); however, prevalence of these methods is not necessarily indicative of water quality. Water quality and access is a major concern in Kejetia. A previous study in Kejetia reported that 100% of households surveyed reported the bush or field as their main toilet facility, compared to only 18.1% of households surveyed throughout Ghana [12]. Further, this study showed high levels of several toxic metals in the community’s drinking water sources, including chromium, manganese, arsenic and lead. This study also reported that 70% of households surveyed in Kejetia (compared to only 14.5% of households nationwide) take greater than 30 minutes to obtain drinking water. 

### 3.2. Characteristics of Survey Respondents

#### 3.2.1. Education

Education is critical in helping individuals make decisions that affect their health. The percentage of women with no education increases in higher age groups in Kejetia (Table S1), and across Ghana, implying that over time, there has been an improvement in education of women. The same is generally true of men. In general, in Kejetia, the percentage of men and women with no education is lower than the national percentage in younger age groups, and higher than the national percentage in older age groups. There is a large difference between the percentage of women with no schooling between those living in the Upper East region (55.0%) *versus* the rest of Ghana (31.3%). The same pattern applies to men, but the overall percentage of men with no education is smaller (43.0% in Upper East and 22.3% in Ghana as a whole) (Table S1). In general, across all age groups, the percentage of women in Kejetia with no education is higher than the percentage of men with no education. These data suggest that women are generally disadvantaged compared to men with regards to education. 

#### 3.2.2. Exposure to Mass Media

Health information is often disseminated through mass media such as radio and television. Like schooling, having access to this information can influence decisions that individuals make regarding their health. In Kejetia, information about mass media exposure was collected in 2010 from only women. Among these women, 35.6% reported listening to the radio at least once per week, compared to 76.4% of women surveyed nationwide in the Ghana DHS. For television, here 23.3% women reported watching television compared to 54.0% surveyed nationwide.

#### 3.2.3. Employment

The 2008 Ghana DHS defined “currently employed” as having done work in the past seven days, not omitting those who did not work in the past seven days but who are regularly employed and were absent from work for leave, illness, vacation, or similar reasons. Nationwide, nearly three-quarters of women and 78% of men surveyed between the ages 15–49 were employed at the time of the 2008 Ghana DHS. This rate is higher in the Upper East for both women (78.3%) and men (80.5%). The employment rate also generally appears to be higher in rural areas (78.8% for women, 81.2% for men) than urban areas (70.5% for women, 74.2% for men).

Our survey categorized interviewees as being “currently employed” if they had done work in the two weeks preceding the survey, or if they still had a job or business even if they had not done work in the past two weeks. In Kejetia, 100% of women and 95.7% of men surveyed between the ages 18-49 were employed at the time of our 2011 survey. 

The 2008 Ghana DHS asked currently-employed respondents to describe their occupation as either professional/technical/managerial, clerical, sales and services, skilled manual labor, unskilled manual labor, or agriculture. Nationwide, 51.4% of women aged 15–49 reported working in sales and services, 10.7% reported doing skilled manual labor, and 29.9% reported working in agriculture. In rural areas and in the Upper East (49.0% and 51.0%, respectively) agriculture was a more common occupation. For men age 15–49 surveyed nationwide, 41.6% reported agriculture as their occupation, followed by skilled manual labor (20.5%), sales and services (12.4%), and professional, technical, and managerial trades (12.2%). The percentage of men involved in agriculture was higher in the Upper East (69.5%) and rural areas (62.4%).

In Kejetia, we categorized currently-employed respondents’ occupations as mining, farming, food vending, some other type of vending or sales, or other. Participants were allowed to describe more than one occupation, as many members of both communities maintained more than one job at a time. In Kejetia, among 47 women age 18 to 75 surveyed about their current occupation, 61.7% reported working in mining, 6.4% reported farming, 31.9% reported food vending, 29.8% reported some other type of vending or sales, and 17.0% reported some other occupation. 

In Kejetia, among 50 men age 18 to 52 surveyed about their current occupation, 88.0% reported working in mining, 8.0% reported farming, 0% reported food vending, 8.0% reported some other type of vending or sales, and 8.0% reported some other occupation. 

#### 3.2.4. Health Insurance

Ghana has prioritized, universal, affordable healthcare coverage, and passed the national health insurance scheme in 2003 with the goal of removing financial barriers to healthcare services [25]. Rates of health insurance coverage among women age 15–49 appear slightly lower in Kejetia (52.4%) than elsewhere in the Upper East (55.1%) but higher than rural (36.8%) and urban (43.2%) areas. Among men in the same age range, these rates are higher in Kejetia (35.3%) than elsewhere in the Upper East (27.9%) or rural (25.3%) and urban (35.9%) areas of Ghana. In general, rates of health insurance coverage are higher for women than for men and higher in urban areas than rural areas. 

#### 3.2.5. Tobacco Usage

Smoking tobacco products and inhaling secondhand smoke increases the risk of cardiovascular disease, lung cancer, and other cancers. It may also increase the severity of respiratory illnesses, such as pneumonia, emphysema, and chronic bronchitis [26]. Children exposed to secondhand smoke may be more vulnerable to disease, particularly respiratory disease, and their growth may be adversely impacted [27]. According to the 2008 Ghana DHS, only 0.2% of women age 15–49 surveyed nationwide used cigarettes, and 0.2% used some other kind of tobacco. In the Upper East, 0.3% of such women used tobacco and none used cigarettes. Among women surveyed in this age category in Kejetia, none reported using any kind of tobacco product. Among men age 15–49, the 2008 Ghana DHS reported that 6.2% of those surveyed nationwide used cigarettes, 0.2% used a pipe, and 1.7% used some other kind of tobacco. In the Upper East, the rates of men using cigarettes, pipe tobacco, and other tobacco were somewhat higher, at 11.6%, 0.8%, and 3.9%, respectively. In Kejetia, 27.7% of men surveyed in that age group reported using cigarettes, and 1.0% used pipe tobacco.

### 3.3. Maternal and Child Health

#### Delivery and Antenatal Care

Antenatal care is critical to maternal and child health as it provides an opportunity for treatment against anemia, as well as malaria and other infectious diseases, screening and treating pregnancy complications, and dispensing information to prepare mothers for delivery. 

In both Kejetia and nationwide, about 3% of women aged 15–49 who had a live birth in the past five years had not received antenatal care for their last birth. More women nationwide (23.5%) than in Kejetia (3.3%) received care from a doctor, and in both groups the majority of women saw a nurse or midwife (Table 2). The percentage of women who received antenatal care from a doctor *vs*. a midwife in Kejetia was more reflective of that in the Upper East than elsewhere in Ghana. In the Upper East, 3.7% of women received no antenatal care, 14.3% received care from a doctor, and 67.9% received care from a nurse or midwife. Another 12.9% received care from community health officers (who provide healthcare services from community-based health planning and services (CHPS) compounds [28]), compared to 6.7% in Kejetia and 5.4% nationwide. Overall, the percentage of women receiving antenatal care from a skilled provider (defined as a doctor, nurse, midwife, or community health officer) was lower in Kejetia (86.7%) than elsewhere in the Upper East (95.7%) or elsewhere in Ghana (95.4%), though the fact that data were missing from 6.7% of women surveyed in Kejetia on the subject may affect these results (Table 2). 

Quality of antenatal care can be assessed by the services provided. Among women with a live birth in the past five years (who may not have necessarily received antenatal care), the percentage who took iron tablets or syrup during the pregnancy for their last birth was higher among women in Kejetia (96.0%) than nationwide (86.5%) and elsewhere in the Upper East region (83.8%). Among women who received antenatal care for their most recent birth in the five years before the survey, 88.9% in Kejetia were weighed, 92.6% had their blood pressure taken, 92.6% had a urine sample taken, and 85.2% had a blood sample taken. These rates appear higher in Kejetia than the rest of the nation, but lower than elsewhere in the Upper East. Nationwide, 97.0% were weighed, 97.1% had their blood pressure taken, 90.3% had a urine sample taken, and 90.4% had a blood sample taken. In the Upper East, 98.7% were weighed, 98.8% had their blood pressure taken, 95.0% had a urine sample taken, and 96.0% had a blood sample taken.

**Table 2:** Percent distribution of antenatal care service providers and person providing assistance during delivery in Kejetia according to our 2011 demographic survey, and in urban Ghana, rural Ghana, and Ghana as a whole according to the 2008 Ghana DHS (Ghana Statistical Service 2009).

Percent distribution of women age 15–49 who had a live birth in the five years preceding the survey by antenatal care (ANC) provider											
	Doctor	Nurse/Midwife	Auxiliary Midwife	Community Health Officer	Traditional Birth Attendant (Trained)	Traditional Birth Attendant (Untrained)	Other	No One	Missing	Total	Percentage Receiving Antenatal Care from a Skilled Provider *
**Residence**											
Urban	33.9	60.1	2.0	1.8	0.3	0.0	0.3	1.4	0.2	100.0	97.8
Rural	16.4	65.3	4.3	7.8	0.2	0.1	1.0	4.8	0.1	100.0	93.9
**Region**											
Upper East	14.3	67.9	0.7	12.9	0.0	0.0	0.0	3.7	0.6	100.0	95.7
**Total**	23.5	63.2	3.4	5.4	0.2	0.1	0.7	3.5	0.1	0.0	95.4
**Kejetia 2010**	3.3	76.7	0.0	6.7	0.0	3.3	0.0	3.3	6.7	100.1	86.7
**Percent distribution of live births in the five years preceding the survey by person providing assistance during delivery**											
**Residence**											
Urban	19.6	61.6	2.6	0.6	8.6	3.5	2.0	1.2	0.4	100.1	84.3
Rural	5.8	32.8	2.3	2.0	20.8	20.5	11.9	3.4	0.5	100.0	43.0
**Region**											
Upper East	1.4	33.7	3.8	7.8	6.5	16.0	27.7	2.2	0.9	100.0	46.7
**Total**	11.0	43.7	2.4	1.5	16.2	14.1	8.1	2.5	0.5	0.0	58.7
Kejetia 2010 **	3.4	48.3	3.4	3.4	0.0	13.8	17.2	0.0	10.3	100.0	58.5

* Skilled provider includes doctor, nurse, midwife, auxiliary midwife, and community health officer; ** includes only the most recent birth in the five years preceding the survey.

According to the 2008 Ghana DHS, nationwide, 57.1% of live births in the five years preceding the survey were delivered in a health facility, compared to only 46.1% of such births in the Upper East. In the Upper East, 33.7% of such women were assisted by a nurse or midwife, 27.7% by another person, and only 1.4% by a doctor (Table 2). In urban Ghana, more women were assisted by a nurse or midwife (61.6%), or a doctor (19.6%). In rural Ghana, a smaller percentage of women were assisted by a nurse or midwife (32.8%), or a doctor (5.8%), and a larger percentage (41.3%) was assisted by a trained or untrained traditional birth attendant. Only 5.8% were assisted by a doctor, and 11.9% were assisted by another person (Table 2). In Kejetia in 2010, among women with live births in the five years preceding the survey, 57.2% had given birth in a health facility for their most recent birth. In terms of assistance received during delivery, 48.3% of women in Kejetia were assisted by a nurse or midwife, 17.2% by a relative or other person, 13.8% by an untrained traditional birth attendant, and 3.4% by a doctor.

### 3.4. Malaria

#### Mosquito Net Ownership and Usage

Mosquito net ownership appears higher in the Upper East region than elsewhere in Ghana, and higher in Kejetia than elsewhere in the uUpper eEast. Only 45.4% of households in Ghana own at least one mosquito net, compared to 55.0% in the Upper East, and 68.5% in Kejetia. The average number of mosquito nets per household is 1.4 in Kejetia, with fewer nets per household in the Upper East and in Ghana (1.0 and 0.7, respectively). Mosquito net ownership generally appears to be more common in Ghanaian rural areas than urban areas. Approximately half (53%) of rural households own at least one mosquito net, with an average of 0.9 nets per household, compared 37% of urban households with an average of 0.5 nets per household.

The percentage of children under five years of age who slept under a mosquito net in the night preceding the interview was higher in Kejetia (73.7%) than in the Upper East (42.9%) or Ghana as a whole (41.1%). A similar pattern was observed for women of child-bearing age as 59.4% of women in Kejetia, 32.0% in the Upper East, and 26% in Ghana as a whole used a mosquito net the night before the interview. It is worth noting that like mosquito net ownership, mosquito net usage the night preceding the survey was higher among children and women in rural areas (45.4% and 35.3%, respectively) than children and women in urban areas (34.2% and 16.5%, respectively). These numbers also suggest that children are generally more likely to sleep under a bed net than women of child-bearing age. Mosquito net ownership and usage was higher in the Upper East than the rest of Ghana due to region-specific initiatives to reduce malaria prevalence, including efforts funded by USAID that aimed to distribute 64,000 nets in remote locations in the region between 2009 and 2012 [29].

### 3.5. Diet and Nutrition

#### 3.5.1. Diet and Nutrition

Adequate nutrition is crucial to child development, and insufficient maternal nutrition is associated with a variety of adverse health outcomes in offspring, such as delayed growth, reduced lung function, impaired cognitive development, and possibly diminished immune function [30]. Maternal iron deficiency before and during pregnancy increases the risk of pre-term birth and low birth weight delivery [31]. Intake of vitamin A during pregnancy and is important to ensure adequate stores to avoid clinical symptoms in the mother and in the fetus [32].

**Table 3:** Percent distribution of foods consumed by women in the day and night preceding the interview in Kejetia according to our 2011 demographic survey, and in urban Ghana, rural Ghana, the Upper East, and Ghana as a whole according to the 2008 Ghana DHS (Ghana Statistical Service 2009).

	Ghana DHS 2008				Survey 2011
	Urban *	Rural *	Upper East *	Total	Kejetia **
Milk	27.9	10.5	15.4	17.2	45.2
Tea/coffee	31.7	14.8	23.1	21.3	16.1
Other liquids (including alcohol)	21.1	12.2	7.7	15.6	19.4
Foods made from grains	88.2	84.7	96.5	86.0	100.0
Foods made from roots/tubers	59.0	69.1	41.9	65.2	9.7
Foods made from legumes	24.2	27.7	35.0	26.4	51.6
Meat/fish/shellfish/poultry/eggs	93.5	84.8	82.7	88.1	100.0
Cheese/yogurt	12.4	4.3	5.4	7.4	0.0
Vitamin A-rich fruits/vegetables	59.2	62.8	95.5	61.4	48.4
Other fruits/vegetables	68.9	62.3	57.2	64.8	25.8
Other solid or semi-solid food	27.5	26.4	70.6	26.8	0.0
Foods made with oil/fat/butter	59.8	47.5	59.4	52.2	74.2
Sugary foods	22.8	12.8	14.6	16.6	16.1
Number of women	571	921	81	1492	31

* Mothers age 15–49 with a child under age three years living with them; ** all women age 15–49 with a child under age three years living with them.

Breastfeeding is an important means of providing necessary nutrients to developing infants and children. The rate of breastfed children born in the five years preceding the survey appears consistent among women in Kejetia in 2010 and the Upper East Region and Ghana’s rural and urban areas in 2008 at about 97%–98%. With regard to micronutrient intake, in the day and night preceding the interview, 100.0% of children age 6–35 months whose households were surveyed in Kejetia in 2011 consumed foods rich in vitamin A, and 92.3% consumed foods rich in iron. Further, in Kejetia, 100% of women age 15–49 living with a child under three years consumed foods rich in vitamin A, and 100% consumed foods rich in iron in the day and night preceding the interview. For both children and women, these numbers are higher in Kejetia than elsewhere in the Upper East and in Ghana as a whole. A dietary survey of women in Kejetia showed lower fruit and vegetable consumption, and higher sugar and fat consumption than other rural areas and other parts of the Upper East (Table 3).

#### 3.5.2. Dietary Diversity

The 2011 survey asked more detailed questions about food consumed in the day and night before the interview (Table 3). Consumption of milk was higher in Kejetia (45.2%) than in the Upper East (15.4%) and nationwide (17.2%). Foods made from grains were consumed by 100% of women in Kejetia, and a smaller percentage of respondents in Ghana as a whole (86.0%). Consumption of roots and tubers was lower in Kejetia (9.7%) than elsewhere in Ghana (65.2%), including the Upper East (41.9%). In Kejetia, 100% of women surveyed had consumed meat, fish, shellfish, poultry, and/or eggs, a higher percentage than women surveyed nationwide (88.1%). Vitamin A-rich fruits and vegetables were eaten by a lower percentage of women from Kejetia (48.4%) than nationwide, (61.4%) and the Upper East (95.5%). Other fruits and vegetables were consumed by fewer women in Kejetia (25.8%) than nationwide (64.8%). 

## 4. Conclusions

This descriptive study found that Kejetia exhibits a number of demographic characteristics that distinguish it from other parts of Ghana. Kejetia’s population structure comprises individuals of working age, with most (both male and female) involved with ASGM and supportive activities. Despite being a productive community in terms of work, Kejetia lacks basic infrastructure that can be found in the rest of Ghana, such as cement flooring, sanitation systems, and piped water sources. Community members in Kejetia reported limited options for potable water sources. Even though households in Kejetia reported more outdoor cooking than other households in Ghana, biomass fuels, especially charcoal and wood, were more common in Kejetia than elsewhere in Ghana, potentially posing a higher risk for adverse health outcomes associated with exposure to smoke from biomass fuel burning. 

Employment rates in Kejetia are high relative to other parts of Ghana and this is driven by the active ASGM sector. The rate of ownership of certain goods that serve as a proxy for household wealth, such as mobile phones and radios, was higher in households surveyed in Kejetia was than that in other communities in rural Ghana, suggesting that disposable income in Kejetia may be higher. However, rates of electricity, television, and refrigerator ownership were lower, and the electricity in Kejetia appeared to be used almost exclusively for mining operations and not for personal use. In Kejetia, the percentage of interviewees with no education was generally higher than that in Ghana as a whole, but generally lower than that in the Upper East. Women surveyed in Kejetia reported less exposure to mass media than households surveyed elsewhere in Ghana. Health insurance rates were lower in Kejetia than in the Upper East as a whole, but higher than in other parts of Ghana, and like elsewhere in Ghana, were higher among women than men. Tobacco usage was higher among surveyed men in Kejetia than among surveyed men in the Upper East or other regions of Ghana.

In Kejetia, certain metrics of maternal and child health care are comparable to those throughout the rest of Ghana, whereas others show deficiencies. Fewer women surveyed in Kejetia receive antenatal care from a skilled provider than similar women elsewhere in Ghana. However, the timing and frequency of antenatal care visits is comparable to that in rural settings, and the rate of important services provided during antenatal is higher in Kejetia than the rest of Ghana. Likewise, women surveyed in Kejetia have similar rates of childbirth in a healthcare facility to other parts of Ghana. In terms of variables related to malaria transmission, mosquito net ownership is higher in Kejetia than elsewhere in the Upper East or in the rest of Ghana. 

Residents surveyed in Kejetia do not appear to be nutritionally deficient in terms of key micronutrients, but did report lower fruit and vegetable consumption and higher sugar and fat consumption than other rural areas and other parts of the Upper East. This may because they have more disposable income to purchase packaged foods or foods prepared by local vendors and, thus, rely less on local agricultural products than other rural communities. 

While by some metrics Kejetia is comparable to other parts of Ghana in terms of factors that influence health, some of our team’s previous research suggests that other factors may indeed be creating health hazards for Kejetia residents. For example, our previous work has shown that several drinking water sources in Kejetia contain coliform bacteria, which may indicate that pollution from human feces may have entered water sources owing to lack of sanitation infrastructure [12]. Lack of clean water sources, such as boreholes or connection to local municipal water supplies, leads many Kejetia residents to drink, cook, and wash with water that contains concentrations of heavy metals above recommended values [12]. 

Further research is needed to determine whether the demographic and health conditions found in Kejetia are similar across ASGM communities in Ghana and elsewhere in the world. Proposed interventions, such as the formalization of the sector, strengthened mining policy, and successful implementation of health programs, technical solutions, and other interventions to improve quality of life for mining communities are dependent upon the availability of robust data [33,34]. 

## Supplementary Files

Supplementary File 1
