# Supplementary material for: Understanding the Social Context of the ASGM Sector in Ghana: A Qualitative Description of the Demographic, Health, and Nutritional Characteristics of a Small-Scale Gold Mining Community in Ghana
Source: Int J Environ Res Public Health. 2015 Oct 12;12(10):12679–96. doi: 10.3390/ijerph121012679 (PMC4626993; doi:10.3390/ijerph121012679)
Supplement: Supplementary File 1 [file ijerph-12-12679-s001.pdf]

Understanding the Social Context of the ASGM Sector in Ghana: A Qualitative Description of the Demographic, Health, and Nutritional Characteristics of a Small-Scale Gold Mining Community in Ghana

Rachel N. Long [1], Elisha P. Renne [2] and Niladri Basu [1,3,*]

Table S1. Percent distribution of educational attainment of the male and female household population in Kejetia according to our 2011 demographic survey.

Background characteristic	No School	Nursery/Preschool	Completed Primary	Completed Midde/JHS	Completed Secondary/SHS, Tech, Voc	Higher than Secondary Education	Missing	Total	Number
**Female (Age)**									
6–9	0.0	80.0	20.0	0.0	0.0	0.0	0.0	100.0	5
10–14	11.1	44.4	44.4	0.0	0.0	0.0	0.0	99.9	9
15–19	29.4	11.8	29.4	29.4	0.0	0.0	0.0	100.0	17
20–24	33.3	11.1	16.7	16.7	16.7	0.0	5.6	100.1	18
25–29	44.4	16.7	22.2	11.1	0.0	0.0	5.6	100.0	18
30–34	27.3	9.1	45.5	9.1	0.0	0.0	9.1	100.1	10
35–39	54.5	0.0	27.3	9.1	9.1	0.0	0.0	100.0	11
40–44	100.0	0.0	0.0	0.0	0.0	0.0	0.0	100.0	3
45–49	100.0	0.0	0.0	0.0	0.0	0.0	0.0	100.0	4
50–54	100.0	0.0	0.0	0.0	0.0	0.0	0.0	100.0	1
55–59	0.0	0.0	0.0	0.0	0.0	0.0	0.0	NA	0
60–64	0.0	0.0	0.0	0.0	0.0	0.0	0.0	NA	0
65+	100.0	0.0	0.0	0.0	0.0	0.0	0.0	100.0	4
**Total**	46.7	16.3	19.3	10.4	3.7	0.0	3.7	100.1	135

Table S1. *Cont.*

Background characteristic	No School	Nursery/Preschool	Completed Primary	Completed Midde/JHS	Completed Secondary/SHS, Tech, Voc	Higher than Secondary Education	Missing	Total	Number
**Male (Age)**									
6–9	0.0	100.0	0.0	0.0	0.0	0.0	0.0	100.0	8
10–14	0.0	40.0	60.0	0.0	0.0	0.0	0.0	100.0	5
15–19	7.7	7.7	38.5	38.5	0.0	0.0	7.7	100.1	13
20–24	5.9	11.8	52.9	11.8	11.8	0.0	5.9	100.1	16
25–29	26.3	0.0	42.1	21.1	10.5	0.0	0.0	100.0	18
30–34	19.0	19.0	23.8	19.0	14.3	0.0	4.8	99.9	21
35–39	21.1	0.0	36.8	26.3	0.0	10.5	5.3	100.0	19
40–44	14.3	0.0	28.6	42.9	14.3	0.0	0.0	100.1	7
45–49	0.0	0.0	50.0	0.0	50.0	0.0	0.0	100.0	2
50–54	66.7	0.0	0.0	0.0	0.0	33.3	0.0	100.0	3
55–59	0.0	0.0	0.0	0.0	0.0	0.0	0.0	NA	0
60–64	0.0	0.0	0.0	0.0	0.0	0.0	100.0	100.0	1
65+	0.0	0.0	0.0	0.0	0.0	0.0	0.0	NA	0
**Total**	29.0	12.3	27.7	18.1	6.5	1.9	4.5	100.0	155

© 2015 by the authors; licensee MDPI, Basel, Switzerland. This article is an open access article distributed under the terms and conditions of the Creative Commons Attribution license (http://creativecommons.org/licenses/by/4.0/).
